# Blood volume-monitored regulation of ultrafiltration in fluid-overloaded hemodialysis patients: study protocol for a randomized controlled trial

**DOI:** 10.1186/1745-6215-13-79

**Published:** 2012-06-08

**Authors:** Manfred Hecking, Marlies Antlanger, Wolfgang Winnicki, Thomas Reiter, Johannes Werzowa, Michael Haidinger, Thomas Weichhart, Hans-Dietrich Polaschegg, Peter Josten, Isabella Exner, Katharina Lorenz-Turnheim, Manfred Eigner, Gernot Paul, Renate Klauser-Braun, Walter H Hörl, Gere Sunder-Plassmann, Marcus D Säemann

**Affiliations:** 1 Department of Internal Medicine III, Nephrology and Dialysis, Medical University of Vienna, Währinger Gürtel 18-20, Vienna 1090, Austria; 2 Malerweg 12, Köstenberg, 9231, Austria; 3 Nikkiso Europe GmbH, Beneckealle 30, Hanover, 30419, Germany; 4 Sozialmedizinisches Zentrum Süd, Kaiser-Franz-Josef Spital, 1st Medical Department, Dialysis, Kundratstrasse 3, Vienna, 1100, Austria; 5 Sozialmedizinisches Zentrum Ost, Donauspital, 3rd Medical Department, Dialysis, Langobardenstrasse 122, Vienna, 1220, Austria

**Keywords:** Dialysis, Ultrafiltration, Renal dialysis, Fluid shifts, Blood volume, Multicenter study, Randomized controlled trials.

## Abstract

**Background:**

Data generated with the body composition monitor (BCM, Fresenius) show, based on bioimpedance technology, that chronic fluid overload in hemodialysis patients is associated with poor survival. However, removing excess fluid by lowering dry weight can be accompanied by intradialytic and postdialytic complications. Here, we aim at testing the hypothesis that, in comparison to conventional hemodialysis, blood volume-monitored regulation of ultrafiltration and dialysate conductivity (UCR) and/or regulation of ultrafiltration and temperature (UTR) will decrease complications when ultrafiltration volumes are systematically increased in fluid-overloaded hemodialysis patients.

**Methods/design:**

BCM measurements yield results on fluid overload (in liters), relative to extracellular water (ECW). In this prospective, multicenter, triple-arm, parallel-group, crossover, randomized, controlled clinical trial, we use BCM measurements, routinely introduced in our three maintenance hemodialysis centers shortly prior to the start of the study, to recruit sixty hemodialysis patients with fluid overload (defined as ≥15% ECW). Patients are randomized 1:1:1 into UCR, UTR and conventional hemodialysis groups. BCM-determined, ‘final’ dry weight is set to normohydration weight −7% of ECW postdialysis, and reached by reducing the previous dry weight, in steps of 0.1 kg per 10 kg body weight, during 12 hemodialysis sessions (one study phase). In case of intradialytic complications, dry weight reduction is decreased, according to a prespecified algorithm. A comparison of intra- and post-dialytic complications among study groups constitutes the primary endpoint. In addition, we will assess relative weight reduction, changes in residual renal function, quality of life measures, and predialysis levels of various laboratory parameters including C-reactive protein, troponin T, and N-terminal pro-B-type natriuretic peptide, before and after the first study phase (secondary outcome parameters).

**Discussion:**

Patients are not requested to revert to their initial degree of fluid overload after each study phase. Therefore, the crossover design of the present study merely serves the purpose of secondary endpoint evaluation, for example to determine patient choice of treatment modality. Previous studies on blood volume monitoring have yielded inconsistent results. Since we include only patients with BCM-determined fluid overload, we expect a benefit for all study participants, due to strict fluid management, which decreases the mortality risk of hemodialysis patients.

**Trial registration:**

ClinicalTrials.gov, NCT01416753

## Background

The importance of volume control has been recognized since the early years of hemodialysis [1], but was previously hampered by the limitations of noninvasive methods used to estimate the hydration state of hemodialysis patients [2]. Recently, the body composition monitor (BCM, Fresenius Medical Care, Bad Homburg, Germany) was introduced, and prescribed to combine a whole-body bioimpedance spectroscopy method with an inbuilt physiologic tissue model [3]. BCM measurements of volume status were successfully validated against gold standard determinations of extracellular and intracellular volume, total body water, fat, fat-free mass and fluid overload [4-7]. Hemodialysis patients with fluid overload >15% extracellular water (ECW), as determined by BCM measurements, were prospectively found to have a markedly higher mortality risk than patients with <15% ECW (hazard ratio (HR) = 2.1, *P* = 0.003) [8]. These results have attracted considerable attention in the dialysis community, and are challenging the concept of interdialytic weight gain (IDWG) as a proxy for fluid retention [9] and measure of nonadherence [10]. By contrast, the novel data imply that reaching the adequate hydration status or ‘dry weight’ postdialysis is more beneficial than reducing fluid intake during the interdialytic interval.

Lowering the dry weight of hemodialysis patients without increasing treatment time can be accompanied by intradialytic complications, mostly cramping and hypotension [11]. Noninvasive hematocrit monitoring technologies, such as crit-line monitoring, were developed as early as 1991 on the basis of optical transmission/optical absorbance [12-15] or ultrasound [16-18] with the ultimate goal to prevent such complications. Determining a hematocrit threshold and manipulating ultrafiltration (UF) rates to maintain the instantaneous hematocrit value two units below the established hematocrit threshold led to a twofold reduction in intradialytic symptoms in hypotension-prone patients [19]. However, various subsequent trials, including the Crit-Line Intradialytic Monitoring Benefit (CLIMB) study [20] failed to yield consistently positive results [21-24], possibly because there is considerable intra- and inter-individual variability of the BVM changes [25].

In the CLIMB study, blood volume monitoring (BVM, for example, crit-line monitoring) was studied as a voluntary adjunct to care, and no algorithms, clinical management advice or instructions were provided for the conventionally monitored patients [20]. However, response options to BVM have been available since the early ’90s [16] and include feedback regulation of ultrafiltration [26] based on stored BVM measurements [27] using fuzzy logic principles [28], and regulation of the dialysate conductivity [29-35]. Feedback control of blood temperature, for example with the body temperature monitor (BTM, Fresenius) [36-38], is an alternative method to prevent intradialytic hypotension.

In the present study, we use BCM measurements to determine a patient’s ideal dry weight and do not use BVM as a diagnostic tool. However, we utilize BVM response options currently available in modern dialysis machines, namely, (1) regulation of ultrafiltration and dialysate conductivity (UCR, Nikkiso Europe GmbH, Hanover, Germany) and (2) regulation of ultrafiltration (Fresenius) in those hemodialysis patients with ≥15% ECW, who are concurrently subjected to systematic increases in ultrafiltration volume, to reach a final dry weight of normohydration weight −7% of ECW. When using the Fresenius machines, we simultaneously employ the BTM to maintain a patient’s original body temperature (hence UTR means ultrafiltration and temperature regulation). Our aim is to assess whether UCR and/or UTR are superior to conventional hemodialysis with respect to frequency of intra- and post-dialytic complications (primary outcome), and improvement of various secondary outcome parameters, possibly related to improved hemodialysis stability and volume status.

## Methods/design

### Hypothesis

In comparison to conventional hemodialysis, regulation of ultrafiltration and dialysate conductivity (UCR) and/or regulation of ultrafiltration and temperature (UTR) will decrease intra- and post-dialytic complications when ultrafiltration volumes are systematically increased in fluid-overloaded hemodialysis patients.

### Objectives and outcome measures

The primary objective is to demonstrate superiority of ultrafiltration and dialysate conductivity regulation (UCR) and/or ultrafiltration and temperature regulation (UTR) over conventional hemodialysis, in preventing intra- and post-dialytic complications, when fluid-overloaded hemodialysis patients receive systematic fluid reduction, to reach a final dry weight of normohydration weight −7% of ECW postdialysis. The primary outcome measure is the total number of hemodialysis sessions per patient that were accompanied, intra- or post-dialytically, by at least one symptom most likely related to fluid withdrawal (as specified in Table1), divided by the number of hemodialysis sessions at risk (as by study protocol: 12 sessions per patient in study phase 1). Both groups, UCR and UTR, will be compared against the conventional hemodialysis group, and afterward against one another, using the two-sided Student’s *t* test

**Table 1:** Complications related to fluid withdrawa

(1)	Intradialytic cramping
(2)	Intradialytic hypotension (>40 mmHg SBP) within 30 minutes: clinically asymptomatic
(3)	Intradialytic hypotension (>40 mmHg SBP) within 30 minutes: clinically symptomatic
(4)	Clinically symptomatic intradialytic hypotension: unspecified drop in SBP
(5)	Unspecified intradialytic complication, most likely related to fluid withdrawal
(6)	Patient-reported postdialysis complication, most likely related to fluid withdrawal

The secondary objectives are:

(a). To demonstrate superiority of ultrafiltration and dialysate conductivity regulation (UCR) and/or ultrafiltration and temperature regulation (UTR) over conventional hemodialysis, in preventing specific intradialytic complications:when fluid-overloaded hemodialysis patients receive systematic fluid reduction, to reach a final dry weight of normohydration weight −7% of ECW postdialysis. The secondary outcome measure for objective (a) is the total number of hemodialysis sessions per patient which were accompanied by the respective symptom most likely related to fluid withdrawal (1 to 6), divided by the number of hemodialysis sessions at risk (as by study protocol: 12 sessions per patient in study phase 1). Both groups, UCR and UTR, will be compared against the conventional hemodialysis group, and afterward against one another, using the two-sided Student’s *t* test.

1. intradialytic cramping

2. clinically asymptomatic, intradialytic hypotension (>40 mmHg drop in systolic blood pressure (SBP) within 30 minutes)

3. clinically symptomatic, intradialytic hypotension (>40 mmHg drop in SBP within 30 minutes)

4. clinically symptomatic, intradialytic hypotension (even if it is not possible to identify a sudden drop in blood pressure, for example, patients may slowly move towards low blood pressure values (f. ex. below 100 mmHg SBP), and report symptoms)

5. unspecified intradialytic symptoms or events, which are most likely related to fluid withdrawal

6. patient-reported postdialysis complication, most likely related to fluid withdrawal (All of the above also listed in Table1)

(b). To demonstrate superiority of UCR and/or UTR over conventional hemodialysis in allowing patients to reach a lower body weight, relative to his/her postdialysis weight at the beginning of study phase 1 (time zero). The secondary outcome measure for objective (b) is the difference in postdialysis body weight from time zero to the end of study phase 1, divided by the postdialysis body weight at time zero.Example: if a patient has a postdialysis body weight of 65 kg at time zero (the last hemodialysis session before the first dry weight reduction), and reaches a postdialysis body weight of 62 kg at the end of the first study phase, his difference in body weight will be 65 kg – 62 kg = 3 kg. Relative to this patient’s postdialysis body weight at time zero, weight reduction from beginning to end of study phase 1 will be 3/65*100 = 4.61%.The relative weight reduction in both groups, UCR and UTR, will be compared against the conventional hemodialysis group, and afterward against one another, using the two-sided Student’s *t* test.

(c). To assess the amount of sodium transferred to the patient or withdrawn from the patient during conventional hemodialysis, UCR and UTR. The amount of sodium removed during hemodialysis is a function of the ultrafiltration volume, the effective diffusion gradient for sodium, and diffusive sodium clearance. The effective diffusion gradient depends on the plasma water - dialysate sodium (DNa) difference, and the Gibbs-Donnan coefficient, the latter being a function of the plasma protein concentration but also being influenced by other ions in the dialysate [39,40]. The assumption that sodium removal by ultrafiltration is equal to the plasma water sodium concentration, multiplied by the ultrafiltration volume, is a simplification [41,42]. Electrolyte balances may also be influenced by the membrane charge [43]. Therefore, the amount of sodium transferred will be measured on the dialysate side. Sodium transfer will be calculated from the difference between the mean sodium concentration in partially collected, used dialysate and the fresh DNa concentration, multiplied by the total amount of dialysate used. Results will be compared to a two-pool sodium model and parameters of the model will be adjusted using the measured transfer data. The above described method is not applicable to the UCR group because the sodium concentration of fresh dialysate is not kept constant during treatment. In order to establish the model, we will measure at least ten patients during three hemodialysis sessions, and due to the formerly stated, we can only use the dialysate from patients in the conventional hemodialysis group and in the UTR group. However, after having established this model, sodium transfer for all patients - conventional, UTR, and UCR - can be calculated using this very model. The amount of sodium transferred to or withdrawn from the patient will be calculated for each patient, according to the model established in the way stated here above. The secondary outcome measure for objective (d), for example, the sodium transferred in both groups, UCR and UTR, will be compared against the conventional hemodialysis group, and afterward against one another, using the two-sided Student’s *t* test.

(d). To assess if stricter volume control by conventional hemodialysis, UCR and/or UTR influences predialysis serum concentrations of (1) C-reactive protein, (2) D-dimer, (3) fibrinogen, (4) troponin T, and (5) N-terminal pro-B-type natriuretic peptide. These proteins are used as read-outs for inflammation (1, 2, 3), coagulation (2, 3), and cardiac function (4, 5) and are routinely determined at all three participating centers. The secondary outcome measure for objective (e), for example, the concentrations of the indicated laboratory parameters in both groups, UCR and UTR, at time zero and at the end of study phase 1, will be compared against the conventional hemodialysis group, and afterward against one another, using the two-sided Student’s *t* test. The individual change in these parameters, from time zero to the end of study phase 1, will be compared likewise.

(e). To assess if stricter volume control in fluid-overloaded patients (using UCR, UTR or conventional hemodialysis) affects the concentration of various proteins that might serve as novel biomarkers (including high-density lipoprotein (HDL)-associated surfactant protein B; HDL-associated serum amyloid A; HDL-associated apoC-II; plasma tryptophan; plasma choline; plasma trimethylamine-N-oxide; and plasma endotoxin). We have recently discovered that various proteins, including those mentioned here above, may render HDL dysfunctional, in hemodialysis patients [44]. Especially with surfactant, we suspect an association with fluid overload. Moreover, plasma tryptophan, plasma choline, and plasma trimethylamine-N-oxide are markers of end-stage renal disease [45]. Since one or several of these proteins or molecules might serve as novel biomarkers, we would like to use the context of the present trial to establish diagnostic assays and analyze potential changes in the serum concentration of these proteins, before and after fluid removal. Endotoxin, found in the cell membranes of gram-negative bacteria, is a biomarker representing the gut flora. Endotoxin has been shown to be associated with inflammation, nutritional status and even mortality in hemodialysis patients [46]. An incremental rise in endotoxin levels has been shown along with the progression of chronic kidney disease, and especially initiation of hemodialysis [47]. It has been suggested that hemodialysis patients might have high endotoxin levels in the blood due to repeated bacterial translocation from the gut during hemodialysis, secondary to intradialytic changes in blood pressure and/or tissue perfusion. An association with chronic fluid overload has not yet been established, but might be suspected, as a consequence of higher ultrafiltration rates and thus decreased intradialytic stability. Here, we will measure endotoxin levels with an amebocyte limulus assay in all study patients, at time zero and at the end of study phase 1. The secondary outcome measure for objective (f), for example HDL-associated serum amyloid A in both groups, UCR and UTR, at time zero and at the end of study phase 1, will be compared against the conventional hemodialysis group, and afterward against one another, using the two-sided Student’s *t* test. The individual change in these parameters, from time zero to the end of study phase 1, will be compared likewise. The results of this secondary endpoint analysis will be published separately from the clinical results of the present study.

(f). To assess if stricter volume control in fluid-overloaded patients (using UCR, UTR or conventional hemodialysis) affects quality of life measures. The secondary outcome measures for objective (g) are the mental component summary (MCS) and physical component summary (PCS) derived from the Kidney Disease Quality of Life Short Form (KDQOL-SF^TM^) [48]. The scale of both summary scores is 0 to 100 (higher indicating better quality of life). MCS and PCS in both groups, UCR and UTR, at time zero and at the end of study phase 1, will be compared against the conventional hemodialysis group, and afterward against one another, using the two-sided Student’s *t* test. The individual change in these parameters, from time zero to the end of study phase 1, will be compared likewise.

(g). To demonstrate superiority of UCR and/or UTR over conventional hemodialysis in reducing dialysis complications when previously fluid-overloaded patients are entering phase 2 and phase 3 of the presented study. In study phases 2 and 3, patients either have to reduce their dry weight further, or else have to maintain their newly reached dry weight. The secondary outcome measures for objective (h) are potentially all of the items described here above, and will be compared against the conventional hemodialysis group, and afterward against one another, in the same fashion as described here above.

(h). To assess which hemodialysis treatment modality will be chosen by the patients at the end of the study. The choice of treatment will be compared as follows: (1) number of patients choosing UCR treatment against number of patients choosing conventional treatment; (2) number of patients choosing UTR treatment against number of patients choosing conventional treatment; (3) number of patients choosing UCR treatment against number of patients choosing UTR treatment. The statistical test for analyses (1), (2) and (3) will be the two-sided chi square test, for each analysis.

#### Superiority definition

For all objectives listed, superiority will be assumed if a statistically significant difference between one group versus another group can be determined. The significance level (alpha) will be set at 0.05. With regard to the primary endpoint, according to our sample size calculation (see below), this study is suited to detect a minimal difference of 10% between groups, if the occurrence of intradialytic symptoms in the conventional group is similar as in a previous study [20].

### Study design

This study is a prospective, multicenter, triple-arm, parallel-group, crossover, randomized, controlled clinical trial. The crossover design serves the purpose of analyzing, in an exploratory form, the secondary endpoints (g) and (h), but does not contribute to the power of the primary endpoint analysis. The reason is that a meaningful crossover analysis would have necessitated that patients revert back to fluid overload, prior to their entering into a new treatment phase; a procedure that we considered unethical.

### Study setting and organizational details

Patient recruitment and follow-up are conducted at the maintenance hemodialysis facilities of the Medical University of Vienna, the Sozialmedizinisches Zentrum Süd in Vienna and the Krankenhaus Donauspital SMZ Ost in Vienna. Baseline demographics from the Medical University of Vienna hemodialysis facility [49] compare very well to international dialysis demographics, as in our recent analysis using data from the Dialysis Outcomes and Practice Patterns study (DOPPS) [50]. Demographics from the other two hemodialysis facilities are not available prior to the start of this study.

**Figure 1:**
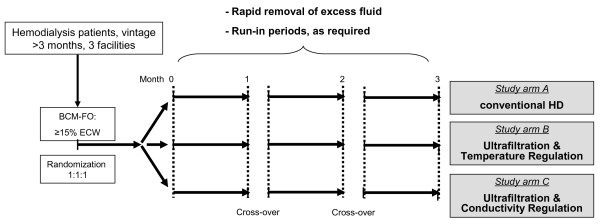
**Study flow chart.** The duration of the crossover period will depend on the run-in phase required for the next study arm: up to six hemodialysis sessions for regulation of ultrafiltration and temperature (UTR), and up to twelve hemodialysis sessions for regulation of ultrafiltration and dialysate conductivity (UCR), no run-in required for conventional hemodialysis. BCM, body composition monitor; ECW, extracellular water.

The study flow chart is depicted in Figure 1. The inclusion criteria are (1) hemodialysis vintage >3 months, (2) fluid overload ≥15% ECW predialysis, as determined by BCM measurement, after one of the two midweek interdialytic intervals, and (3) informed consent of the patient. There are no specific exclusion criteria, other than the reciprocal inclusion criteria, and those criteria implied by good clinical practice and the Declaration of Helsinki (for example, exclusion of minors and of mentally disabled patients). Patients will only be included once. Monitoring of the participants will be ended after the participants’ completion of the study phases.

For patients who are willing to participate in the study, dry weight reduction is prescribed according to a previously published algorithm [11] described here below. Block randomization takes place in a 1:1:1 ratio into study group A (UCR), study group B (UTR) or study group C (conventional hemodialysis). The randomization code is developed using an internet-based randomization tool [51] and is not revealed to participants or investigators. We are stratifying by hemodialysis center, thus there is a different randomization list for each center.

UCR and UTR cannot be applied without previous knowledge of a particular patient’s blood volume changes during the hemodialysis session, which have been shown to be variable, intra- and inter-individually [25]. Therefore, UCR and UTR require a run-in period, which has to be performed every time that UCR and/or UTR are being used. Completion of the run-in period is judged successful for UCR, respectively UTR, if the responsible technicians from Nikkiso, respectively Fresenius, have set the blood volume corridors, respectively have determined the critical point, for adequate use of UCR, respectively UTR. No more than a maximum of twelve, respectively six, hemodialysis sessions are provided to the Nikkiso, respectively Fresenius, technicians, as previously agreed.

Baseline is defined as the day of the BCM measurement. Time zero is defined, for every study phase, as the first hemodialysis session with dry weight reduction after the run-in phase. After completion of the first study period (twelve hemodialysis sessions for all three groups), patients crossover to the respective other study arms, as prespecified at the time of randomization. There is no explicit washout period between the first study phase and any of the subsequent study phases. However, another run-in period is required for the nonconventional treatment arms, before study phases 2 and 3. BCM measurements to adjust or re-prescribe dry weight reduction will be performed before every run-in period, and shortly before time zero of every study phase. If there is reason to believe that patients may have a change in their dry mass during the study period (for example, patients who are hospitalized or who change their diet), BCM measurement will be repeated, and the dry weight adjusted accordingly.

### Study interventions

This study does not interfere with the principle of best practice hemodialysis treatment, which has been described elsewhere [52]. Patients with hemodiafiltration protocols who agree to participate are switched to hemodialysis for the duration of the study.

#### Dry weight reduction

The BCM measurement for fluid overload is provided in kilograms and percentage of ECW. A patient’s new dry weight is calculated as follows: (Predialysis body weight – (fluid overload + 7% ECW)). Thus it is the normohydration weight −7% ECW, and serves as the new, BCM- determined, ideal, or ‘final’ dry weight. In order to reach this ‘final’ dry weight, the patient’s previous dry weight is decreased as previously described in the ‘Dry-Weight Reduction in Hypertensive Hemodialysis Patients’ (DRIP) trial [11]: by 0.1 kg per 10 kg body weight at every hemodialysis session until the final dry weight is reached or any intradialytic complications (listed in Table1) may occur.

**Figure 2:**
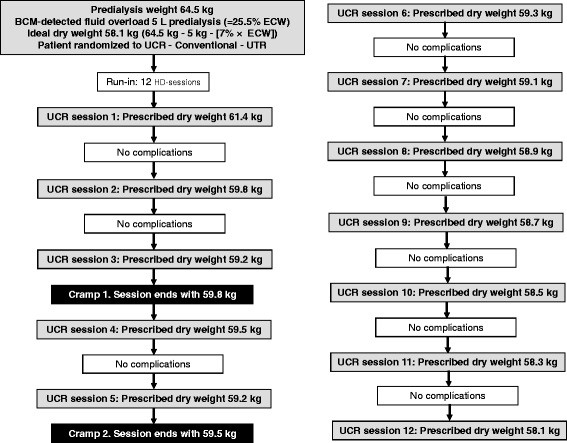
An exemplary dry weight reduction scenario.

An exemplary dry weight reduction scenario is provided in Figure 2. In the event of the first intradialytic complication, standardized clinical interventions take place as listed in Table2, and the dry weight of the ongoing hemodialysis session is re-adjusted to the dry weight of the previous hemodialysis session. At the subsequent hemodialysis session, the dry weight is prescribed as 50% of the previous, additional fluid removal, and all further dry weight reductions are prescribed accordingly. In the event of the second intradialytic complication, standardized clinical interventions take place, and the dry weight at the ongoing hemodialysis session is re-adjusted to the dry weight of the previous hemodialysis session, as before. At the subsequent hemodialysis session, fluid removal is further reduced by 50% of the corrected, additional fluid removal, or to a minimum of 200 g (whatever occurs first, depending on the overall weight of the patient). In the event of a third intradialytic complication, standardized clinical interventions take place, and the dry weight at the ongoing hemodialysis session is re-adjusted to the dry weight of the previous hemodialysis session, as before. No further attempts are made to reduce the dry weight during this study period, unless the patient did not yet reach reduction steps of 200 g.

**Table 2:** Clinical interventions

	**1**^**st**^**intervention**	**2**^**nd**^**intervention**	**3**^**rd**^**intervention**	**4**^**th**^**intervention**
Cramping	20 mL NaCl (1 M)*	according to doctor's order		
Nausea	Trendelenburg position	250 mL NaCl (0.9%)	plasma expander	according to doctor's order
Vomiting				
Clinically symptomatic IDH				
Decrease in intradialytic SBP ≥40 mmHg				
Unspecified	according to doctor's order			

*5.85% weight/volume. *IDH* intradialytic hypotension, *M* molar, *NaC1* sodium chloride, *SBP* systolic blood pressure.

At the occurrence of symptoms, ultrafiltration is decreased and dry weight adapted, as per the study’s protocol.

Justification for the duration of the study phase: 12 hemodialysis sessions per study phase imply that, if regulated ultrafiltration can prevent intra- and post-dialytic complications, a 70 kg patient is, in theory, able to remove as much as 12 × 700 g = 8.2 L of excess fluid within one study phase. Therefore, we expect that for the great majority of patients, we would theoretically be capable of removing all excess fluid within one study phase.

#### UCR and UTR versus conventional hemodialysis

The principle difference between the two techniques under investigation, UCR and UTR, in comparison to the standard treatment (conventional hemodialysis), is that the ultrafiltration rates in the UCR and UTR groups are variable, and - usually - increased at the beginning of the dialysis treatment, when blood volume slopes are flatter, indicating that refilling takes place from the interstitial to the intravascular compartment. By contrast, ultrafiltration rates in the conventional hemodialysis group are constant, and can be calculated by dividing the total amount of ultrafiltration by the dialysis treatment time.

UCR: dialysis machines from Nikkiso which allow UCR are currently in use at all three participating centers. After recording several (up to 12) hemodialysis sessions, an individual BVM reference corridor - above and below the typical blood volume slope - is determined for every patient. Ultrafiltration as well as dialysate conductivity regulation are started from there on until the end of this study period. In addition to variable ultrafiltration rates, the conductivity component of UCR - through intradialytic changes in dialysate sodium - is aimed at further increasing the refilling process, whenever blood volume slopes indicate that this is necessary.

UTR: dialysis machines from Fresenius which allow UTR are currently in use at all three participating centers. After recording several (up to six) hemodialysis sessions, the ‘critical point’ (the lowest point of the BVM curve) is determined. Above this point, the machine may increase ultrafiltration rates during the beginning of the hemodia-lysis session (as by the inbuilt ultrafiltration regulation device). When the critical point is approached, the machine decreases ultrafiltration rates, to avoid intradialytic complications. Ultrafiltration and temperature regulation (by activating the BTM in the temperature control mode) is started from there on until the end of this study period. The temperature component of UTR is aimed at keeping the body temperature constant, and thereby thought to increase intradialytic stability by preventing overheating and thus vasodilation.

Conventional hemodialysis: in the conventional dialysis arm, we do not allow changes in dialysate temperature, but continue with the dialysate temperature of the patient’s last treatment. We also do not allow changes in the DNa prescription from the patient’s last treatment and changes in other aspects of the dialysate fluid composition (this also applies to the UCR and UTR study arms).

Other than ‘intelligent’ regulation of ultrafiltration, the potential advantage of UCR and/or UTR over conventional hemodialysis is that the dialysis machine alarm goes off in the case that the blood volume slope of the patient during a regulated treatment approaches the corridors (UCR/critical points (UTR)) set for that patient. The dialysis nurse then has the possibility to overrule this alarm, or to decrease and/or interrupt the total ultrafiltration prescribed for that particular treatment. Since the aim of UCR and UTR is to decrease dialysis complications, nurses are instructed never to overrule an alarm, but to decrease and/or interrupt the prescribed ultrafiltration.

#### Antihypertensives

Antihypertensives are withdrawn as in a previous study on dry weight reduction [11], starting one week prior to the first hemodialysis session with reduced dry weight. Alpha and beta blockers are withdrawn first, followed by calcium channel blockers and blockers of the renin-angiotensin-aldosterone system last. The accepted systolic blood pressure during the course of the patient’s antihypertensives withdrawal phase shall generally not surpass 200 mmHg.

### Data retrieval and data recording

Intra- and post-dialytic complications (primary outcome parameter) as specified in Table1 are recorded on the patient’s case report form, and filled out at every hemodialysis session. Secondary outcome parameters: the body weight reached at the end of every hemodialysis session is recorded in the patient’s case report form. Residual renal function is assessed by measurements of 24-hour urine. Quality of life forms are kept in paper format and analyzed using MS Excel 2003. All nonexperimental laboratory data are extracted from the patients’ hospital records and analyzed separately from the dialysis-related outcomes using MS Excel 2003.

### Definition of intradialytic and postdialytic complications

The list of intradialytic complications was chosen in close analogy to a list of secondary outcome measures in the CLIMB study (Table 4 in [20]), and to the DRIP trial (Supplementary Table1 in [11]). As an operational defi-nition of what is ‘clinically symptomatic’, we specify that, nausea, dizziness, vomiting, or any other symptom that the patient may report, if it is most likely related to fluid withdrawal, shall be judged as ‘clinically symptomatic’. Based on our dialysis experience and using the DRIP trial reports as a basis [11], we preclude that besides cramping, intradialytic hypotension is the major obstacle in intensified fluid withdrawal, since hospitalizations - also related to vascular access problems - are dealt with in the analysis of serious adverse events. There are no measurements taken against intradialytic hypertension during the course of the study, unless specifically ordered by the responsible physicians, based on individual patient needs.

### Informed consent

The investigator explains the nature of the study, its purpose, procedures, expected duration, and the potential risks and benefits associated with study participation along with any discomfort that may be expected. Patients are informed about the strict confidentiality of their subject data, but also that their medical records may be reviewed for trial purposes by authorized individuals other than their treating physician. Each subject is informed that study participation is voluntary and withdrawal is possible at any time during the study period. Withdrawal does not prejudice the subject’s subsequent care. Subjects are given time to read and understand the statements before signing consent and dating the document. Subjects receive a copy of the signed written statement and the original copy of the informed consent is stored in the investigator study files. No subject is entered into the study until informed consent has been obtained.

### Safety assessments

Safety assessments include the monitoring and recording of all adverse events (AE), including serious adverse events (SAE). An AE is any undesirable experience associated with the use of a medical product or procedure in a patient. An SAE is defined as any untoward medical occurrence that, at any dose, results in death, is life-threatening, requires inpatient hospitalization or prolongation of existing hospitalization, or results in persistent or significant disability/incapacity. The most probable AEs caused by dry weight reduction are cramps and hypotension. The expected intra- and post-dialytic symptoms are provided in Table1. The most probable serious adverse events caused by dry weight reduction are vascular-access-related hospitalizations, and aggravated consequences of postdialysis hypotension, such as fractures due to falling.

### Quality control

Execution of the three techniques under investigation is crucial. Evaluation of the success of UCR and UTR will be evaluated at the end of the first study phase for all available treatments and will include at least the following parameters:

Availability of a reference line for the setting of corridors (UCR) critical values (UTR) during the run-in phase. Specifically, the number of treatments during the run-in phase with an available reference line will be reported per patient, and per all of the treatments during the run-in phase.

Delayed start of UCR/UTR during the study phases. Specifically, the number of treatments during the study phase with a delayed start (>10 minutes) of UCR/UTR will be reported per patient, and per all of the treatments during the study phase.

Delayed input of the ultrafiltration goal during the study phase. Specifically, the number of treatments during the study phase with a delayed input of the ultrafiltration goal (>10 minutes) will be reported per patient, and per all of the treatments during the study phase.

Incorrect input of the ultrafiltration goal during the study phase. Specifically, the number of treatments during the study phase with an incorrect input of the ultrafiltration goal will be reported per patient, and per all of the treatments during the study phase.

Inadequate display of the ultrafiltration rate and conductivity during the study phase. Specifically, the number of treatments during the study phase with an inadequate display of the ultrafiltration rate and conductivity during the study phase will be reported per patient, and per all of the treatments during the study phase.

### Statistical analysis plan

The statistical analysis plan (SAP) provides full details regarding the analyses, the data display, and the algorithms to be used for data derivations. The SAP includes the definition of major and minor protocol deviations, which will be identified by medically trained staff before the study closure. Safety and tolerability are analyzed descriptively. Safety analysis is performed on the intention-to-treat (ITT) population. All secondary endpoint analyses are exploratory in nature.

Sample size calculation: for the primary endpoint analysis, we will assess the differences in intradialytic complications between the three study groups, only during the first study phase. Based on a typical occurrence of 0.14 dialysis-related complications per patient-day at risk in the conventional hemodialysis group [20], two-sided testing and an expected standard deviation of 10%, an alpha = 0.05 and a beta = 0.2, a sample size of 17 patients per group was determined to detect a minimum difference of 10% between groups. The UCR and UTR groups will be tested against the conventional hemodialysis control group, and afterward against one another. We expect a drop-out rate of approximately 10%. Individuals who do not complete the study cannot be replaced, because we do not include patients with hemodialysis vintage <3 months, and all patients who qualify for the study are asked to participate after the initial BCM measurement at all three centers. Thus, the study sample was determined to consist of 60 patients with ECW ≥15%, by BCM measurement. If patients are lost to follow-up, they will be excluded from the analysis

Two different analysis sets are defined for safety and efficacy, respectively. The efficacy of UCR and UTR will be assessed in all subjects who received the study method (at least once) and did not violate the protocol in a way that might affect the evaluation of the effect of the study method on the primary objective, that is, without major protocol violations. The per-protocol set is employed in the analysis of efficacy variables. The safety analysis set includes subjects who were randomized and received at least one study method (modified intention-to-treat). The safety set is employed in the analysis of tolerability and safety variables. Statistical analysis is performed with SAS.

### Approval of the ethics committee and the regulatory authority

The trial is performed in accordance with the Declaration of Helsinki. It subscribes to the principles outlined in the most recent version of the International Conference on Harmonization on Good Clinical Practice. Approval was obtained from the ethics committee of the Medical University of Vienna (EK number 365/2011) and from the ethics committee of the City of Vienna (EK 11-222-1211). The study has also been registered in a public clinical trial database (Identifier Number NCT01416753, ClinicalTrials.gov).

## Discussion

Recent data show prospectively that fluid control based on BCM measurements and proper adjustment of dry weight is feasible [53]. Moreover, normohydrated patients from a German hemodialysis facility did not have a higher multivariate-adjusted, all-cause mortality risk than patients undergoing ‘long, slow hemodialysis’ in Tassin, France [54]. Last, the first prospective experiences with the intervention of BCM-based correction of volume status (for example, lower dry weight prescription) support the mortality benefit of normohydration, shown in the previous association study, where fluid status was assessed once, using BCM, and patients were thereafter followed over three and a half years [8] As a consequence, we expect a direct benefit for all patients participating in this study, because lower dry weight will be prescribed in all three study groups.

The results of the CLIMB study [20], as well as experiences from previous clinical trials, have indicated that BVM is complicated, and have made its use controversial. For example, Andrulli *et al*. found no critical BVM level for the appearance of symptomatic hypotension in either normotensive, hypotension-prone or hypertensive patients [21]. On the contrary, Barth *et al*., upon using an ultrasonic method for BVM, demonstrated an individual relative blood volume limit for nearly all patients, with only narrow variability in most patients prone for intradialytic morbid events [22]. The results of Tonelli *et al*. suggested in patients with acute renal failure that BVM and rate of change in BVM slopes did not predict hypotension and were not correlated with mean arterial pressure or SBP [23]. And finally, Mitra *et al*. observed that the relative blood volume decline during ultrafiltration switched from exponential to linear decay, probably indicating vascular refilling, and suggesting that BVM should move away from linearity [24]. The present study, however, by using prespecified response options to BVM is intended to overcome these previous controversies.

Withdrawal of antihypertensives, in order to reach lower dry weight, may, to some dialysis care takers, seem unethical. However, blood pressure treatment in hemodialysis patients is highly controversial. Although blood pressure control may be the most extensively studied area in dialysis research, widely accepted propositions for the treatment of hypertension are still missing [55,56], mainly because epidemiologic studies have failed to incriminate hypertension as a cardiovascular risk factor [57]. Oral pharmacotherapy has recently been shown to yield some mortality benefit [58,59], but data from single centers in Uruguay [60], United Kingdom [61], Turkey [62] and Tassin, France [63] have consistently demonstrated that strict volume control with relatively low postdialysis dry weight targets can fully normalize the blood pressure in a great majority of hemodialysis patients. In fact, hypertension control without antihypertensive medication is actually the strongest predictor of survival in hemodialysis patients [64].

Therefore, it is ethical to withdraw antihypertensives in order to reach the adequate dry weight target, and this approach has been used before, in the DRIP trial, which did not include patients based on bioimpedance-based detection of fluid overload, but based on hypertension [11]. The mortality risk for the hemodialysis population - in observational studies - does not begin to rise before very high SBP values of 200 mmHg are reached [65,66]. However, we intend not to generally accept that the SBP during the course of the patient’s antihypertensives withdrawal phase surpasses 200 mmHg.

We have recently shown that higher DNa prescriptions are associated with IDWG, but not with worse outcomes, and that predialysis serum sodium concentrations correlate inversely with mortality risk [49,67]. Among secondary outcome parameters, principal emphasis will be placed on a measurement of intradialytic sodium balance (listed as secondary objective (c)). This analysis is necessary, as there is only one previous publication, which estimated, but did not measure sodium transfer through positive dialysate-to-serum sodium gradients [68]. The results of our investigation may help dialysis physicians in their decision concerning the dialysate sodium prescription, because they will be able to anticipate which amount of sodium is transferred during hemodialysis.

As it has been shown that increased cardiovascular biomarkers such as N-terminal pro-B-type natriuretic peptide, D-dimer and troponin T are strongly correlated with inflammation as well as higher cardiovascular mortality in hemodialysis patients [69-71], it will be impor-tant to assess whether systematic dry weight reduction improves the chronic inflammatory state associated with hemodialysis. Combining BCM-based fluid status assessment with UCR and/or UTR-guided hemodialysis, and simultaneous monitoring of these biomarkers, can potentially show changes in the individual patient’s cardiovascular risk profile.

Role of dialysis caretakers; subjectivity: dialysis nurses must be reminded that it is not in the best interest of the patients enrolled in the BVM-Reg study to have fluid withdrawn ‘no matter what’. This is also the reason why we created symptom category (5) (in Table1), the ‘unspecified intradialytic complication’, and category (6), the ‘patient-reported postdialysis complication’, both ‘most likely related to fluid withdrawal’. These categories shall ensure that enrolled patients are maximally protected from discomfort, since they can report necessary occurrences, even postdialysis. In consequence, fluid withdrawal rates are reduced at the subsequent hemodialysis sessions.

Based on our working experience, the great majority of dialysis nurses are aware of the importance of symptom-free hemodialysis sessions, since discomfort is almost always reported to the nurses first. However, how can it be ensured that patients are monitored in the same way? It is impossible to rate the subjective opinion of the patient and his/her primary dialysis caretaker regarding the need to interrupt the fluid withdrawal, using the reported category (5). In addition, it would have been very unrealistic to successfully blind the treatment (UCR, UTR, conventional hemodialysis). The lack of blinding, in combination with the above-mentioned subjectivity of patients and nurses, can introduce bias into the study. By instructing dialysis caretakers to remain as objective as possible toward the treatments under investigation, we anticipate that our study will still yield informative results.

In conclusion, the present study will test the hypothesis that UCR and/or UTR may lead to fewer complications, intra- and post-dialytically, when hemodialysis patients with fluid overload ≥15% ECW receive systematic, and presumably optimal reduction of their dry weight (to the normohydration weight −7% of ECW postdialysis). To the best of our knowledge, this is the first study that combines bioimpedance measurements for assessment of volume status and BVM response options. As a consequence, patients may benefit from two entirely unrelated concepts in modern fluid management, with potential impact not only on intradialytic stability, but also dialysis-associated factors such as residual renal function, inflammation, and quality of life. Ultimately, applying the dialysis treatment that proves superior in the present study may translate into improved dialysis outcomes.

## Trial status

At the time of manuscript submission, the present study - overall - is still recruiting participants (ClinicalTrials.gov, NCT01416753). While participants are being recruited at the maintenance hemodialysis facility of the Sozialmedizinisches Zentrum Süd in Vienna, the study is ongoing at the hemodialysis facility of Krankenhaus Donauspital SMZ Ost in Vienna, and at the hemodialysis facility of the Medical University of Vienna.

## Abbreviations

BCM: Body composition monitor; BTM: Body temperature monitor; BVM: Blood volume monitoring; CLIMB: Crit-Line Intradialytic Monitoring Benefit (study); DRIP: Dry-Weight Reduction in Hypertensive Hemodialysis Patients (trial); DNa: Dialysate sodium; DOPPS, Dialysis Outcomes and Practice Patterns Study; ECW: Extracellular water; HDL: High-density lipoprotein; HR: Hazard ratio; IDH: Intradialytic hypotension; IDWG: Interdialytic weight gain; M: Molar; MCS: Mental component summary; NaCl: Sodium chloride; PCS: Physical component summary; SAE: Serious adverse event; SBP: Systolic blood pressure; UF: Ultrafiltration; UCR: Regulation of ultrafiltration and dialysate conductivity; UTR: Regulation of ultrafiltration and temperature.

## Competing interests

This academic study is sponsored by the Medical University of Vienna, Austria. The authors received an unrestricted research grant from Nikkiso Ltd. with no limits on publication and declare that they have otherwise no competing interests.

## Authors’ contributions

MH, MA and MS are responsible for all aspects of this trial; in particular, they designed the study, wrote and revised the manuscript, are registering study patients and are surveying the study at the Chronic Hemodialysis Facility 1 of the Medical University of Vienna. WW and TR are surveying patients at the Chronic Hemodialysis Facility 2 of the Medical University of Vienna, and revised the present manuscript. JW, MH and TW designed the secondary endpoint analyses listed under (f), and revised the present manuscript. H-DP contributed intellectually to all technical aspects of this study, designed the secondary endpoint analysis listed under (d), and revised the present manuscript. PJ advised us on, and planned the technical aspects of UCR, and also wrote the part on quality control. IE, KL-T, and ME are registering and surveying patients at the hemodialysis facility of Sozialmedizinisches Zentrum Süd. GP and RK-B are registering and surveying patients at the hemodialysis facility of Krankenhaus Donauspital SMZ Ost. WHH and GS-P planned and revised the study protocol, as well as the present manuscript. All authors read and approved the final manuscript.
